# Synthetic Genetic Targeting of Genome Instability in Cancer

**DOI:** 10.3390/cancers5030739

**Published:** 2013-06-24

**Authors:** Babu V. Sajesh, Brent J. Guppy, Kirk J. McManus

**Affiliations:** Manitoba Institute of Cell Biology, Department of Biochemistry and Medical Genetics, University of Manitoba, Winnipeg, Manitoba R3E 0V9, Canada; E-Mails: babu@cc.umanitoba.ca (B.V.S.); umguppyb@cc.umanitoba.ca (B.J.G.)

**Keywords:** cancer, genome instability, tumor suppressor gene, oncogene, synthetic genetic approach, synthetic lethality, synthetic dosage lethality, cancer therapy

## Abstract

Cancer is a leading cause of death throughout the World. A limitation of many current chemotherapeutic approaches is that their cytotoxic effects are not restricted to cancer cells, and adverse side effects can occur within normal tissues. Consequently, novel strategies are urgently needed to better target cancer cells. As we approach the era of personalized medicine, targeting the specific molecular defect(s) within a given patient’s tumor will become a more effective treatment strategy than traditional approaches that often target a given cancer type or sub-type. Synthetic genetic interactions are now being examined for their therapeutic potential and are designed to target the specific genetic and epigenetic phenomena associated with tumor formation, and thus are predicted to be highly selective. In general, two complementary approaches have been employed, including synthetic lethality and synthetic dosage lethality, to target aberrant expression and/or function associated with tumor suppressor genes and oncogenes, respectively. Here we discuss the concepts of synthetic lethality and synthetic dosage lethality, and explain three general experimental approaches designed to identify novel genetic interactors. We present examples and discuss the merits and caveats of each approach. Finally, we provide insight into the subsequent pre-clinical work required to validate novel candidate drug targets.

## 1. Introduction

Cancer is a leading cause of death worldwide. In 2008, the World Health Organization (WHO) estimated that ~7.6 million deaths worldwide were attributable to cancer, a number that represents ~13% of all deaths [1]. The WHO also predicted that the number of cancer-related deaths will rise to ~12 million individuals per year by 2030 [1]. Many current chemotherapeutic strategies often involve the systemic administration of a drug whose cytotoxic effect is incapable of distinguishing cancerous from normal cells. Many common chemotherapeutics affect DNA replication or cellular division and predominantly impact cancer cells due to their rapid proliferation rates relative to normal, non-cancerous cells. As a result, adverse side effects do occur within normal cell types undergoing replication, particularly those of the hematopoietic lineages. Consequently, novel therapeutic strategies and drug targets are urgently needed that enable the targeted killing of cancer cells to decrease adverse side effects while minimizing the morbidity and mortality rates associated with cancer.

Synthetic genetic interactions that reveal pathway interactions have been studied extensively in model organisms for decades and are now being explored for their therapeutic value in human cancer contexts. Over the past decade, a growing body of evidence suggests that synthetic genetic approaches may be successful in delivering enhanced targeting and killing of cancer cells, and therefore may hold therapeutic potential for many tumor types. Conceptually, these approaches seek to exploit the aberrant genetics (e.g., mutation, deletion or amplification) associated with tumor development, and are predicted to evoke highly specific killing of cancer cells while minimizing side effects within normal cells. Synthetic genetic targeting of tumor cells represents a paradigm shift from traditional approaches and can be generally classified into two distinct categories; (1) synthetic lethal approaches that specifically exploit the aberrant genetics and epigenetics associated with hypomorphic expression and/or function typically within tumor suppressor genes; and (2) synthetic dosage lethal approaches that are designed to exploit the aberrant genetics and epigenetics associated with hypermorphic expression and/or function typically within oncogenes. Below is a basic description of genome instability, its relevance to cancer, and how it can be exploited through synthetic genetic approaches for enhanced specificity and targeting of cancer cells. Following this are descriptions and examples of how synthetic lethal and synthetic dosage lethal approaches are designed to identify novel candidate drug targets. Included within each section are brief descriptions of the types of tests and screens that can be performed to identify novel synthetic genetic interactions including some of the benefits and caveats associated with each.

## 2. Discussion

### 2.1. Therapeutically Exploiting the Molecular Origins of Genome Instability

Somatic mutations [2,3,4] and epigenetic changes [5] underlying genome instability are recognized as significant predispositions that drive tumorigenesis. Consequently, the genetic and epigenetic insults that are associated with tumor development may be the very targets that can be exploited through synthetic genetic strategies. Genome instability is associated with virtually all tumor types, including both solid and liquid tumors, and generally occurs through three mechanisms; (1) Microsatellite Instability (MIN), which is defined by an increase in basal mutation rate and stems from defects in the DNA mismatch repair pathway [6,7,8,9,10,11]; (2) CpG Island Methylator Phenotype (CIMP), which is most frequently associated with the epigenetic silencing of tumor suppressor genes due to DNA hypermethylation within promoter regions [5,12,13,14]; and (3) Chromosome Instability (CIN), which is defined by an increase in the rate at which numerical and/or structural chromosome aberrations occur [3,15,16,17,18]. Recent and extensive DNA sequencing efforts have identified a myriad of tumor-specific mutations, deletions, and amplifications in hundreds of candidate genes involved in a number of biological pathways that affect genome stability including DNA repair, DNA replication, and chromosome segregation [19,20,21,22,23,24,25,26,27]. Subsequent functional analyses will be required to identify those alterations that have functional consequence. However, most pertinent to a synthetic genetic targeting strategy is the fact that these mutations or epigenetic modifications distinguish cancer cells from normal cells. Thus, synthetic genetic approaches are custom-tailored to the specific aberrant genetic and epigenetic phenomena that occur within cancer cells, and their therapeutic effect(s) and killing are fundamentally restricted to cancer cells.

Genetic and epigenetic changes that drive CIN are particularly attractive targets for synthetic genetic approaches—CIN is frequently observed in tumors and its presence correlates with poor patient outcome [28,29,30,31,32,33,34,35,36]. Conceptually, CIN promotes tumor heterogeneity by decreasing or increasing chromosome numbers or structural rearrangements, which impacts tumor suppressor gene and oncogene copy numbers. In general, most solid tumors arise through the accumulation of genetic and epigenetic defects within tumor suppressor genes and proto-oncogenes that encode loss-of-functions and gain-of-functions, respectively [37]. Proteins encoded by tumor suppressor genes such as *TP53* [38] and *RB1* [39] normally function to preserve genome stability. They generally function by limiting cell cycle progression and proliferation so that normally occurring errors in DNA can be appropriately repaired. As a result, diminished expression and function are associated with an increase in genome instability and thus they are excellent targets for therapeutic intervention. On the other hand, enhanced or ectopic expression and function of proto-oncogenes (e.g., *ERBB2* [*HER2*/*NEU*] [40,41,42], *MYC* [43,44,45], and *RAS* [46,47,48]) causes aberrant growth factor/mitogenic signaling and accelerates cell cycle progression. Oncogenic alterations also promote cell survival by inducing anti-apoptotic mechanisms particularly within cellular contexts (e.g., genome instability) where it would normally be induced (see [49]). Consequently, targeting the aberrant etiological origins, such as altered tumor suppressor genes and/or oncogenes that cause genome instability may be an effective way to selectively restrict the therapeutic targeting to cancer cells.

The synthetic genetic targeting of aberrant tumor suppressor genes and/or oncogenes represents an evolution from traditional therapeutic approaches in two critical ways. First, synthetic genetic approaches do not specifically target the aberrant gene *per se*, but rather exploit the defect by targeting a second unlinked gene partner (*i.e.*, a synthetic genetic interactor). In principle, only cancer cells harboring specific defects will be susceptible to a synthetic genetic attack, and thus all normal cells will remain unaffected. Second, synthetic genetic approaches are broader in scope as they are capable of targeting either tumor suppressor genes or oncogenes. Most conventional strategies are designed to combat the gain-of-function associated with oncogenes (e.g., *ERBB2* [41]), and all but ignore tumor suppressor genes due to the inherent complexities in restoring a loss-of-function(s) mutation within a tumor cell. Furthermore, it may now become possible to develop combinatorial strategies that simultaneously target both tumor suppressor genes and oncogenes within a given tumor. This approach would not only enhance the targeting of cancer cells and minimize side effects, but may also produce a synergistic cytotoxic effect within the cancer cells. Thus identifying and characterizing synthetic genetic interactors of both tumor suppressor genes and oncogenes are critical steps for the development of the next generation of candidate drug targets and therapeutic strategies.

### 2.2. Synthetic Lethality

**Figure 1:**
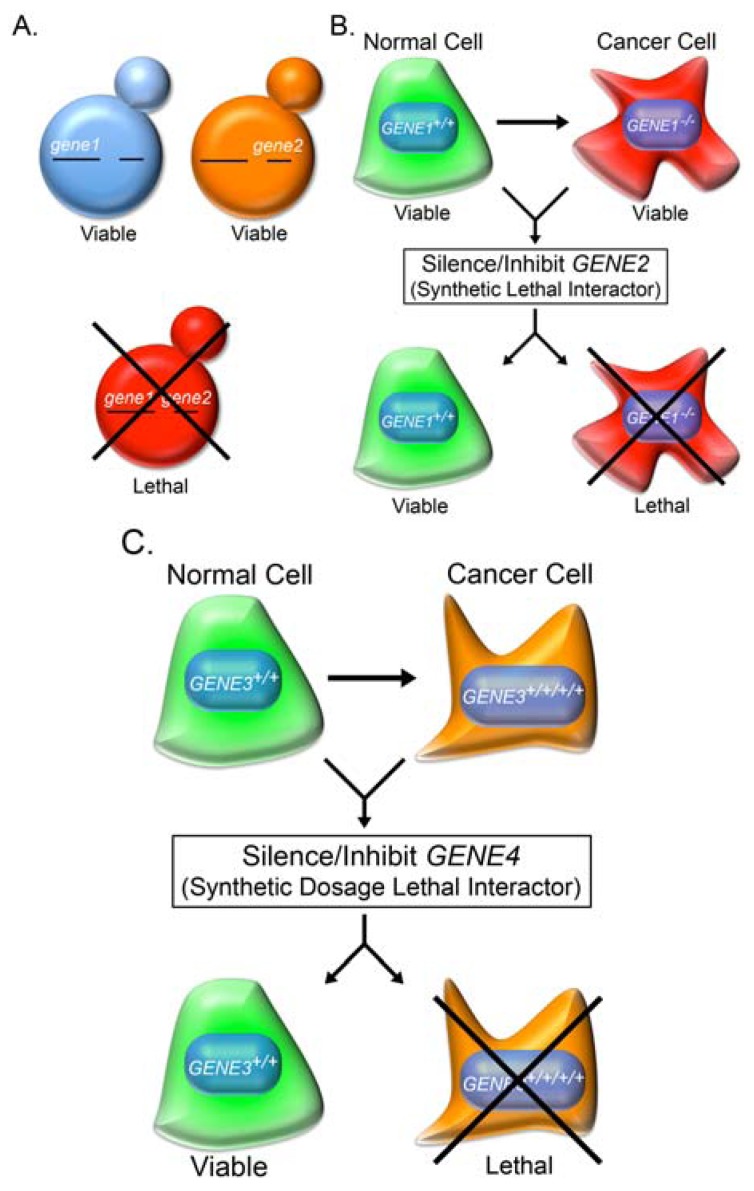
Synthetic Genetic Approaches in Model Organisms and Cancer. (**a**) Synthetic lethality is a rare genetic interaction that occurs when two independent and viable mutations or deletions (*gene1* [blue yeast] or *gene2* [orange yeast]) result in death when combined (red yeast). If a slow growth phenotype is observed, a synthetic growth defect or synthetic sickness is defined. (**b**) A cancer-associated hypomorphic mutation or deletion in a gene (e.g., *GENE1* is a deleted tumor suppressor gene) is selectively killed through a synthetic lethal approach by silencing or inhibiting the protein product encoded by *GENE2*. (**c**) A cancer-associated hypermorphic mutation or amplification (e.g., *GENE3* is an amplified oncogene) is selectively killed through a synthetic dosage lethal interaction by silencing or inhibiting the protein product encoded by *GENE4*.

**Figure 2:**
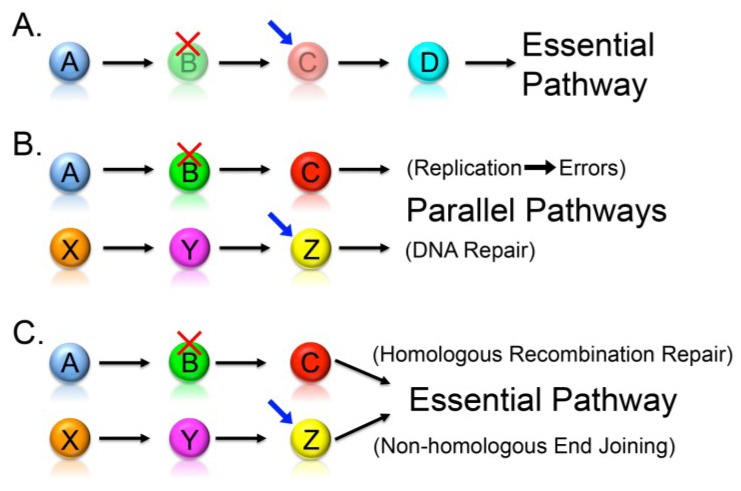
Conceptual Models Depicting the Underlying Mechanisms that Account for Synthetic Lethal Interactions. Conceptual models detailing three mechanisms that produce a synthetic lethal interaction—circles represent genes, cancer-associated mutations, or deletions are identified by a red “X”, and synthetic lethal interactors (*i.e.*, drug targets) are identified by blue arrows. (**a**) Partial ablation of two functions encoded within a single essential pathway (e.g., epistasis group), such that the pathway is no longer functional. (**b**) Ablation of two functions encoded within two distinct parallel pathways. For example, defects in DNA replication would lead to DNA errors requiring repair, and small molecule inhibitors preventing accurate repair will cause lethality. (**c**) Ablation of two functions encoded within two separate pathways that together impinge on a single essential process. For example, DNA double strand breaks can only be repaired through two pathways, namely homologous recombination repair and non-homologous end joining—defects and inhibition of both pathways will cause cellular cytotoxicity.

In 1946, Theodosius Dobzhansky, a geneticist and evolutionary biologist, first coined the term synthetic lethality to describe a lethal genetic interaction observed when two independently viable homologous chromosomes were allowed to recombine in *Drosophila pseudoobscura* [50]. Synthetic lethality is now used to describe a rare and lethal genetic interaction in which the outcome of a particular mutation or deletion is influenced by the presence of a pre-existing mutation or deletion (Figure 1). However, if slowed growth rather than death is observed, a synthetic growth defect or synthetic sickness is defined. Synthetic lethal interactions generally occur via three basic mechanisms and are depicted in Figure 2; (1) partial ablation of two proteins contained within the same essential biological pathway, or epistasis group such that the pathway becomes non-functional; (2) ablation of two proteins contained within parallel pathways both of which are required for viability; and (3) ablation of two proteins within parallel pathways that together impinge on an essential biological pathway or process. This approach can be extrapolated to a cancer context (see [51] and Figure 1B) where a somatic mutation in a gene normally required to maintain genome stability represents a sensitizing mutation that will render all subsequent progeny susceptible to attack by down-regulating or inhibiting a synthetic lethal interactor [52,53].

In 1997, Hartwell and colleagues [54] suggested that cancer cells harboring somatic mutations represent genetically sensitized cells, relative to normal surrounding cells that may be susceptible to drug therapies by selectively targeting a synthetic lethal interactor [55,56]. The development of both RNAi-based libraries and gene knockout models in a variety of model systems, coupled with significant advances in high-content and high-throughput approaches has now made it possible to screen, identify and validate synthetic lethal interactors in a variety of model organisms and cancer-model systems. In fact, many researchers are now routinely employing these reagents, resources and approaches to identify novel candidate drug targets by uncovering therapeutically exploitable synthetic lethal interactors of genes that are somatically mutated in various tumor types. In general, synthetic lethal interactors have been identified through three independent, yet interrelated approaches that predominantly differ only in scale and scope. These approaches include: (1) knowledge-based direct tests; (2) cross-species candidate gene approaches; and (3) whole genome-based approaches. These three strategies, including both their merits and caveats, are discussed below.

### 2.3. Knowledge-Based Direct Tests to Identify Synthetic Lethal Interactors

Decades of focused biochemical and genetic research in many model organisms and systems coupled with the recent advancements in RNAi-based libraries and gene knockout models have significantly increased our knowledge about the biological components and processes that impact genomic stability. Many critical players within these pathways are well characterized including members involved in DNA replication, DNA repair and mitogenic signaling. Many research programs are now focused on determining how aberrant expression and/or function of these pathway members contribute and drive the development and progression of cancer. For example, a number of extensive gene sequencing efforts have been conducted in various tumor types (e.g., colon, breast, lung, *etc.*) that have identified somatic alterations in a large number of genes that normally encode functions within pathways required to maintain genome stability [19,20,21,22,23,24,25,26,27]. These mutations include non-synonymous single nucleotide polymorphisms (nsSNPs) encoding single amino acid substitutions or premature stop codons, and gene amplifications and deletions that depending on the gene context (*i.e.*, oncogene *vs.* tumor suppressor gene) may be associated with hypermorphic or hypomorphic expression and/or function, respectively. Candidate synthetic lethal interactors of somatically altered tumor suppressor gene mutations can be identified through knowledge-based approaches, which relies on fundamental knowledge about the molecular players and biological processes in which they participate. Knowledge-based tests are therefore predictions of synthetic lethal interactors based on *a priori* knowledge of the molecular constituents of pathways and molecular network whose members are mutated in cancer.

The prototypic and perhaps best-studied example of a knowledge-based approach is the synthetic lethal interaction observed between *BRCA1* (or *BRCA2*) and *PARP1* (poly ADP-ribose polymerase 1). *BRCA1* and *BRCA2* are classically defined as breast cancer susceptibility genes but each is also somatically altered in a number of tumor types including ovarian, prostate and colon [21,22,24,25]. BRCA1 and BRCA2 both function in homologous recombination repair, which is critical for “error-free” repair of DNA double strand breaks (reviewed in [57]). PARP1 on the other hand, is involved in single strand break repair, and has traditionally been shown to function within the base excision repair pathway [58,59], however, more recent data indicate it may not be involved with base excision repair, but single strand break repair, nonetheless [60]. PARP1 consists of evolutionarily conserved functional domains designed to detect single-stranded DNA damage and elicit a response in the form of ADP-ribose polymerization (reviewed in [61,62]). Armed with the above information and the knowledge that single-strand breaks can be converted to double-strand breaks during DNA replication [63], two research teams reasoned that PARP1 inhibition would cause single strand breaks which when left unrepaired would ultimately lead to double-strand breaks that could not be efficiently repaired within the *BRCA1*- or *BRCA2*-defective backgrounds [64,65]. Indeed as predicted, targeted killing of the *BRCA1*- and *BRCA2*-defective cells occurred following PARP1 inhibition and/or silencing relative to controls, which was further substantiated in embryonic stem cells [65] and animal models [64], thus validating PARP1 as a candidate drug target in *BRCA1* and *BRCA2*-defective cells.

The initial characterization of a synthetic lethal interaction between *BRCA1*/*BRCA2* and *PARP1* spawned a number of subsequent small molecule inhibitor screens and studies to identify novel PARP1 inhibitors [66]. PARP1 inhibitors generally fall into two categories: (1) competitive binding to the catalytic domain, which prevents substrate binding, or (2) non-competitive binding, which can disrupt complex formation and thus adversely impact function. Many PARP1 inhibitors (e.g., Veliparib, CEP-9722, Rucaparib, E7016, BMN-673, *etc.*) are now being evaluated both as single agents and in combinatorial approaches in a number of tumor types (see [67]), and Olaparib (KuDOS Pharmaceuticals; KU-0059436 or AstraZeneca; AZD-2281) is perhaps the best-known PARP1 inhibitor in clinical trials (see [68] for current clinical trial updates). Olaparib is an orally active compound that inhibits PARP1 through competitive binding with NAD+ and the first clinical report was published in 2009 [69]. Early phase I clinical trials in breast cancers with *BRCA*-deficiencies suggested that Olaparib was tolerated and may exhibit a beneficial clinical response [70], and a subsequent Phase II clinical trial was initiated in women with advanced stage breast cancer [71]. The clinical cohort was divided into two groups with the first group receiving a high dose Olaparib (400 mg, 2-times daily) and the second group a low dose (100 mg, 2-times daily). The overall response rate within the first group was 41% *vs.* 22% for the second group, suggesting that Olaparib, and PARP1 inhibition, may hold therapeutic promise. Another putative PARP1 inhibitor, Iniparib (Sanofi-Aventis) was reported to be a non-competitive inhibitor that made it through Phase I and Phase II clinical trials for breast cancer, but it eventually failed a Phase III trial [72,73]. However, the ultimate failure of Iniparib may not be due to the synthetic lethal strategy employed, but rather to the compound itself as two independent research teams failed to find evidence for significant PARP1 inhibition following Iniparib treatments in cell-based assays [74,75] suggesting that Iniparib may not be a potent PARP1 inhibitor. There is still considerable interest in both competitive and non-competitive PARP1 inhibitors as therapeutic agents in cancer treatments. In fact, there are at least a dozen clinical trials in various stages evaluating Olaparib as a single or combinatorial agent in a variety of tumor types (e.g., breast, ovarian, prostate, *etc.*), with numerous other inhibitors in various stages as well (see [67]). Thus, the *BRCA1/BRCA2 PARP1* synthetic lethal interaction and PARP1 inhibitors in general, are still being evaluated for their therapeutic potential. However, their ultimate clinical success will have to be determined through these continued clinical trials.

**Figure 3:**
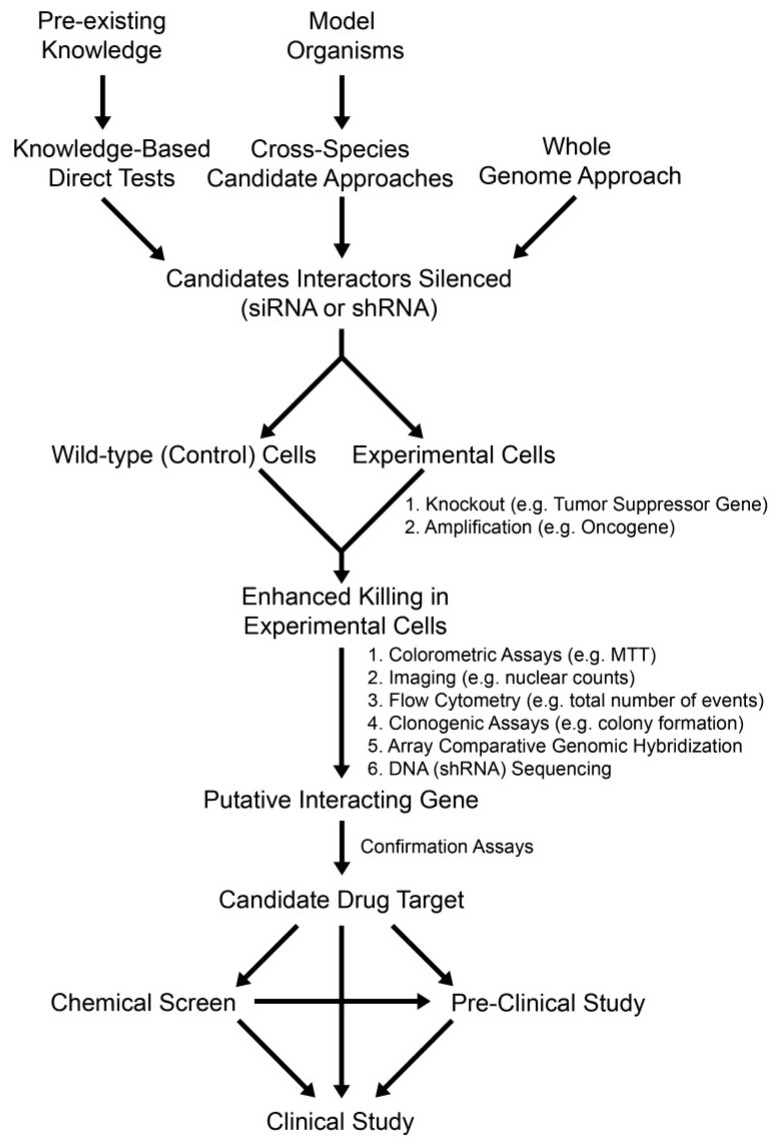
Fundamental Approaches to Identify Synthetic Genetic Interactors. A flowchart depicting the three fundamental experimental approaches designed to identify synthetic genetic interactors. Knowledge-based direct tests, cross-species approaches or whole genome approaches can be employed to screen for candidate interactors (*i.e.*, drug targets) of a given cancer query gene. Depending on the scale of the screen either siRNA duplexes or shRNA constructs can be employed so that direct comparisons can be made between the experimental query line and an isogenic control line. In general, gene knockout cells or gene amplification cells are employed as the query lines for tumor suppressor genes (synthetic lethal interactors) and oncogenes (synthetic dosage lethal interactors), respectively. Targeted killing of the query cell line relative to an isogenic control is typically evaluated using any one of a number of experimental assays—six common assays are listed. Once a putative interactor is identified, subsequent validation is required before it is confirmed as a novel candidate drug target. Depending on the target, a chemical screen may be initiated, which may yield candidates requiring subsequent validation, optimization and pre-clinical study. Alternatively, if known inhibitors exist they can be evaluated in either pre-clinical studies, or, if the compound has already received approval for use in other human diseases, clinical studies may be initiated with appropriate approval.

To expand beyond the prototypic synthetic lethal example described above, a similar knowledge-based approach can be employed to identify novel synthetic lethal interactors that can exploit genetic defects occurring specifically within tumor suppressor genes (Figure 3). There is currently a wealth of biochemical and genetic data available for a large number of biological processes in which many synthetic lethal interactors are likely to exist. As indicated above, the pathways and members involved in both single [76] and double [77,78] strand DNA break repair are relatively well characterized (see [79]). In fact, many constitutive and conditional gene knockout models exist for pathway members, which are frequently mutated in various tumor types that are ideally suited to synthetic lethal testing. Isogenic models of tumor suppressor genes (*i.e.*, cancer query genes) are excellent systems to employ for testing as they have genetically identical backgrounds. Because these cell-based models only differ by a single gene they are well suited to evaluate highly specific interactions occurring between a given cancer query gene and a candidate synthetic lethal interactor. Most importantly, the knockout cells act as a surrogate for the variety of pathogenic gene alterations that occur within tumors including homozygous gene deletions, epigenetic silencing and certain nsSNPs that confer hypomorphic expression and/or function. Consequently, these isogenic models can be employed to test specific synthetic lethal interactions by down-regulating or inhibiting the expression of a candidate interactor through RNAi or small molecule inhibitors, respectively, and assaying for enhanced death within the knockout cells. Conceptually, if a synthetic lethal interaction occurs, a decrease in cell numbers will be apparent within the knockout cells relative to the wild-type controls. Differences in cell numbers can be easily identified through a variety of established approaches (Figure 3) that are routinely employed or available in many laboratories including colorimetric assays (e.g., 3-(4,5-dimethylthiazol-2-yl)-2,5-diphenyltetrazolium bromide [MTT]), fluorescence microscopy (nuclear counts), flow cytometry (total number of events), real-time cellular analyses (growth curves) or longer-term clonogenic assays (colony formation). In fact, many of these assays can be multiplexed with live/dead cell indicators that will facilitate the simultaneous characterization of a targeted cytotoxic effect within the knockout cells. In any case, the putative synthetic lethal interactors identified through these direct tests will require thorough validation to confirm them as novel candidate drug targets. Once validated, these candidates will undergo small molecule inhibitor screens and rigorous pre-clinical studies to further evaluate their efficacy and therapeutic potential prior to initiating clinical trials.

The knowledge-based approach described above is not limited to DNA repair pathway members and theoretically can be expanded to include any biological pathway. However, our ability to identify and evaluate synthetic lethal interactors will be limited by our current understanding of the specific proteins that function within those pathways. Beyond the caveats associated with the silencing efficacy of RNAi-based approaches and specificity concerns associated with small molecule inhibitors, there are additional factors that will impact the success of a knowledge-based approach. These factors may include cell- and tissue-specific gene expression profiles as well as genetic and functional redundancy within certain pathways. For example, if a candidate interactor is not normally expressed in a particular tissue, or alternatively a genetically redundant gene is expressed that functionally compensates for the candidate interactor, a lethal phenotype will not occur. Finally, the cellular contexts in which these experiments are performed such as transformed *vs.* immortalized cell types, or combinatorial approaches involving chemotherapeutics that induce genotoxic stress, will undoubtedly impact gene expression profiles that may influence synthetic lethal interactions. Nevertheless, this approach has been successfully applied (see *BRCA1 PARP1* above) and suggests it will likely identify many additional synthetic lethal interactors.

### 2.4. Cross-Species Candidate Gene Approaches to Uncover Conserved Synthetic Lethal Interactors

Since the original description in flies, synthetic lethal approaches have been expanded to include studies in numerous genetically tractable model organisms including worms and yeast. As stated above, Hartwell and colleagues [54] suggested that cancer cells harboring somatic mutations may be susceptible to drug therapies that selectively target a synthetic lethal interactor. They also argued that synthetic lethal interactions identified in model organisms could be used to identify conserved interactors in humans. Since the molecular basis of many essential biological processes particularly those required to maintain genome stability are highly conserved throughout evolution, it is likely that synthetic lethal interactions will also be conserved. Thus synthetic lethal datasets generated in model organisms can be mined to identify candidate interactors to evaluate in human cancer contexts.

The most extensive synthetic genetic studies to date have employed the budding yeast deletion mutant arrays (collections of ~4,700 non-essential gene deletion strains) to systematically interrogate all pair-wise gene combinations to produce comprehensive synthetic genetic interaction networks [80,81,82,83]. Over the past decade these efforts have provided novel insight into biological function and pathway architecture, and have helped define molecular complex and epistasis group members [80,81,82,83]. Perhaps the greatest unrealized attribute of these networks lies in their ability to predict candidate conserved synthetic lethal interactions. Although previous studies by Lehner *et al*. [84] and Byrne *et al*. [85] suggest there is limited conservation between yeast and worms, these studies were limited to signaling cascade members not implicated in genome/chromosome stability. However, more recent studies interrogating members of known genome and chromosome stability pathways such as the spindle assembly checkpoint [86] and the alternative replication complex [87] indicate that up to 50% of synthetic lethal interactors are conserved. Additional support for conserved interactions comes from Dixon *et al*. [88] who identified significant overlap in the synthetic lethal networks of two distantly related eukaryotes. More recently and most relevant to humans, McManus *et al*. [52] employed a cross-species approach to identify the first conserved synthetic lethal interaction in a cancer context that was first identified in budding yeast. Using siRNA-based silencing and high-content imaging they showed that diminished FEN1 (flap endonuclease 1) expression induced cellular selective cytotoxicity within *RAD54B*-deficient colorectal cancer cell but not in isogenic control cell line. RAD54B is normally involved in homologous recombination repair [89] and somatic mutations are found in numerous tumor types [22,24,25,26,90]. A subsequent study expanded this initial proof-of-principle to include five additional genes, which are frequently mutated in cancer (*CDC4*, *MRE11A*, *RNF20*, *SMC1A*, and *SMC3*) that are also selectively killed following FEN1 inhibition [53]. These data indicate that FEN1 is synthetic lethal with numerous cancer mutated genes and further validate FEN1 as a candidate drug target. Next, a fluorescence-based assay was developed and employed to screen a chemical library composed of 30,000 compounds for novel FEN1 inhibitors. Of the 13 candidate inhibitors identified with *in vitro* activity, three exhibited *in vivo* activity within two different cell-based models [53]. These three compounds now represent lead small molecule inhibitors that require further pre-clinical testing and subsequent optimization prior to initiating human clinical trials.

A major benefit of the cross-species approach is that it can potentially accelerate the discovery and validation of novel candidate drug targets. Synthetic lethal interactions are arguably best characterized in budding yeast owing to their genetic tractability and the availability of deletion mutant arrays. As a result, large synthetic lethal datasets exist for budding yeast (and other organisms) that are publically available through online resources including the Biological General Repository for Interaction Datasets [91,92,93,94] (BioGRID; [95]). These genetic data can be accessed from that database or alternatively displayed using a genetic data visualization tool such as Osprey [96] or Cytoscape [97]. Because synthetic lethal interactions are rare genetic events, most yeast query genes typicallyremo exhibit fewer than 130 synthetic lethal interactions (out of a possible ~6,000 pair-wise combinations). Consequently, if human homologs are identified, small RNAi-based screens can be performed to interrogate a highly focused set of candidate interactors (typically ranging up to 100 candidate interactors) in a human cancer context. In principle, these screens will be similar to those described within the knowledge-based approach and will employ isogenic cell lines. As indicated above, any putative interactors identified will require extensive validation and pre-clinical work prior to confirming them as a novel candidate drug targets (Figure 3).

As with a knowledge-based approach, there are certain caveats associated with a cross-species approach. First of all, much of the current synthetic lethal interaction data comes from budding yeast, which harbor ~6,000 total genes compared to ~20,000 in humans. Consequently, any cross-species approach involving yeast (or any other model organism) mandates there be a homologous human protein, whether it be the cancer query gene or the candidate synthetic lethal interactor. For example, the role of aberrant TP53 and RB1 expression in cancer is well established and synthetic lethal interactors that selectively kill tumors cells with these defects would be of tremendous benefit. Unfortunately, yeast do not have *TP53* or *RB1* homologs and thus a cross-species approach involving yeast cannot be performed. However, homologs do exist in other model organisms (e.g., flies) and thus synthetic lethal datasets from these model systems can be employed as guides in a human cancer context. A second caveat is that there is a requisite expansion in the total number of genes within humans that reflects the fundamental genetic difference from the single cell eukaryote. Contained within the expanded complexity is an additional layer of genetic redundancy and evolution that is associated with cell and tissue differentiation that has the potential to impact synthetic lethal interactions in the human context. Nevertheless, the genetic basis and molecular players involved in many essential biological processes, such as those required to maintain genome and chromosome stability (e.g., DNA repair, replication and segregation) are evolutionarily conserved, and cross-species candidate gene approaches have been successfully employed to identify conserved synthetic lethal interactors. Accordingly, synthetic lethal datasets derived from model organisms represent highly valuable repositories that will help accelerate the identification of synthetic lethal interactors that will ultimately become candidate drug targets for subsequent small molecule inhibitor screens and pre-clinical studies.

### 2.5. Whole Genome RNAi-Based Screens to Identify Synthetic Lethal Interactors

Whole genome approaches are also being employed to uncover novel candidate drug targets by identifying synthetic lethal interactors for any given cancer gene query. However, unlike the knowledge-based and cross-species approaches detailed above that limit the number of potential interactors interrogated, a whole genome approach represents an unbiased survey of all possible interactors. Consequently, whole genome approaches are expected to identify additional synthetic lethal interactions beyond those identified by the above approaches (Figure 3). Whole genome based screens also differ from the other two approaches in two specific ways. First, the total number of genes evaluated is significantly larger and approaches gene saturation. The shRNA libraries are purposefully designed to include multiple constructs targeting unique regions of the same gene. Consequently, shRNA libraries often contain ≥60,000 unique shRNA constructs. This is critical as silencing efficiencies can vary between different constructs targeting the same gene. However, having multiple constructs targeting a single gene can also be beneficial. For example, if similar results are obtained with multiple shRNAs, a candidate interactor can be quickly validated. Second, because the shRNA construct must stably integrate within the host genome, retro-viral (*i.e.*, lenti-viral) based approaches are employed. To ensure that only a single gene is silenced and interrogated in any given cell, a low multiplicity of infection (MOI < 1) is typically used. Furthermore, whole genome approaches are typically performed in a pooled manner where cells are transduced *en masse* with shRNA constructs. It is not possible to perform this type of whole genome approach using traditional siRNA-based approaches due to the vast number of genes interrogated in a given screen and the difficulty in ensuring only a single gene is targeted within a given cell. These features are critical as either DNA sequencing or microarray analyses are ultimately performed (rather than the cell-based assays employed in the sections above) to identify those shRNA constructs that become depleted from the cellular pool over time, which presumably occurs due to cellular cytotoxicity induced by a synthetic lethal interaction. As with the siRNA-based approaches detailed above, any candidate synthetic lethal interactors identified using a whole genome approach requires subsequent validation prior to confirming it as a novel candidate drug target.

The development of shRNA-based libraries and the advancement of next generation sequencing platforms have enabled individuals to perform genome-wide synthetic lethal screens [98,99,100]. For example, Xie *et al*. [101] recently performed a whole genome screen to identify synthetic lethal interactors of *TP53*. Isogenic wild-type and *TP53*-deficient colorectal cancer cells (HCT116) were transduced with an shRNA library containing approximately 60,000 clones. Genomic DNA was isolated from cells 40 hours and 10 days post-transduction, PCR amplified and the presence of shRNA constructs was confirmed through DNA sequencing. The disappearance of an shRNA construct at the 10-day time-point relative to the 40-hour time-point specifically within the TP53-deficient cells was suggestive of a synthetic lethal interaction. Based on this screen a total of 103 candidate synthetic lethal genes were identified, from which a subset were subsequently validated including ETV1, a member of the ETS family of transcription factors, and ATR, a DNA damage checkpoint protein*.*

The fundamental benefit of a whole genome RNAi-based screening strategy is that it is an unbiased approach that will uncover synthetic lethal interactors for any given query gene. As stated above, it should identify not only the synthetic lethal interactors identified through the two approaches detailed above, but all synthetic lethal interactors for a given query gene. However, depending on the statistical threshold employed, the number of targets requiring validation can be increased or decreased accordingly. This will minimize and limit subsequent analyses to only the best candidates (*i.e.*, those that are depleted the most), but may also eliminate a potentially superior therapeutic target that is not efficiently silenced by the shRNA. As with the knowledge-based and cross-species approaches, whole genome strategies also come with certain caveats. First and foremost, the ability of a primary screen to identify putative synthetic lethal interactors depends solely on the ability of the library to efficiently silence each of the ~20,000 human genes, and it is known that the shRNA libraries do not offer complete (*i.e.*, 100%) coverage for all annotated genes. In addition, the level of silencing achieved can be heterogeneous for the genes that are targeted in the library. For example, the silencing of some targets is exceptional, while others do not appear to be silenced at all, and due to the scale of this work, it is not possible to validate the silencing efficiency of all targets through conventional approaches (e.g., RT-PCR or Western blots). The cost and access to critical infrastructure required to execute and analyze these screens can be a major factor for many laboratories considering whole genome approaches. Nevertheless, these approaches have been successfully undertaken and have identified large numbers of candidate interactors. Thus, it is likely that as costs diminish, these approaches will become more attractive and will yield large numbers of putative synthetic lethal interactors that will require subsequent validation through direct tests.

### 2.6. Synthetic Dosage Lethality

Oncogenic transformation is a critical step in the tumorigenic process as it provides a selective growth advantage that drives the development and progression of cancer. However, it may also represent an Achilles heel that can be selectively targeted through synthetic dosage lethality. In 1996, Kroll and colleagues [102] developed a variation of the traditional yeast synthetic lethal screen in which they showed increasing the expression or activity of a protein could produce a lethal phenotype in a genetically sensitized, mutant strain. This concept, termed synthetic dosage lethality was based on previous observations in yeast in which increased toxicity was observed following gene overexpression in specific mutant strains. Overexpression of *MCM3* (mini-chromosome maintenance 3) for example, exacerbates defects observed in *mcm2* mutants [103], while *ORC6* (origin of replication complex 6) overexpression lowers the non-permissive temperature associated with *cdc46-1* mutants [104]. Thus, synthetic dosage lethality has long been established in single cell eukaryotes and may hold therapeutic potential in a human cancer context. Indeed, a large number of oncogenes (e.g., *ERBB2*, *MYCN*, *KRAS*, *etc.*) are amplified at the level of the genome within various tumor types and this amplification not only underlies hypermorphic expression and/or function, but also serves to differentiate the tumor cells from normal surrounding tissues. Thus, the gain-of-function associated with oncogenes, rather than the loss-of-function associated with tumor suppressor genes, may also be therapeutically exploited by uncovering synthetic dosage lethal interactors. Consequently, the search for novel candidate drug targets that can exploit gain-of-function mutations within oncogenes in humans has begun.

Although the three synthetic lethal approaches detailed above are designed to identify novel candidate drug targets that exploit genetic defects in tumor suppressor genes (*i.e.*, hypomorphic expression/function), it is not difficult to envision how they can be modified to identify targets that exploit the genetic defects associated with hypermorphic expression and/or function of oncogenes (Figure 3). Indeed, two studies showed that synthetic dosage lethality occurs in humans and may be an effective therapeutic strategy to target cancers harboring specific oncogenic mutations. For example, Molenaar *et al*. [105] recently demonstrated that silencing of CDK2 preferentially induced apoptosis within a neuroblastoma cells harboring three copies of *MYCN* (3 × *MYCN*), but not within 1 × *MYCN* lines or in fibroblast controls. *MYCN* (or *N*-Myc) is a proto-oncogene that normally encodes a transcription factor involved in regulating cell cycle progression, apoptosis, cell proliferation and neurogenesis [106,107] and it is overexpressed in neuroblastoma, medulloblastoma, and Wilm’s tumor [108,109,110,111,112]. They subsequently showed that *MYCN* silencing within the 3 × *MYCN* line abrogated the apoptotic response indicating that MYCN overexpression is essential to induce synthetic dosage lethality. In 2009, Luo and colleagues [113] performed a genome wide screen to uncover synthetic dosage lethal interactors of an oncogenic *KRAS* mutant (*KRAS^WT/G13D^*) in colorectal cancer cells. In essence, the increased dosage occurred as a result of hypermorphic function associated with KRAS^G13D^ expression. KRAS is a GTPase that normally functions in growth factor signaling and hypermorphic *KRAS* mutations occur frequently in various tumor types including colon, lung and uterine cancer [19,25,114,115]. Initially, a total of ~1,600 candidate *KRAS* interactors were identified that were subsequently narrowed to ~380 candidates when a more stringent statistical cutoff was applied. Subsequent validation assays determined that 77 interactors could selectively impact the fitness of the *KRAS^WT/G13D^* mutant cells relative to controls including numerous proteins involved in mitotic progression, the anaphase promoting complex/cyclosome and the proteasome. Consequently, a relatively large number of candidate drug targets were identified that warrant further study. The above two examples serve to illustrate that synthetic dosage lethal approaches can be performed and novel candidate drug targets can be identified that can specifically exploit hypermorphic expression and/or function of oncogenes.

The central tenet of a synthetic dosage lethal approach is that it exploits the very molecular defects that drive tumor development—hypermorphic expression and/or function of oncogenes. Thus, testing and validating synthetic dosage lethal interactors is critical to uncover novel candidate drug targets that can selectively target these genetic defects. In essence, similar, albeit slightly modified screens to those listed within the synthetic lethal sections can be employed to identify synthetic dosage lethal interactors. For example, an oncogenic query cell line (e.g., amplified or hypermorphic mutation) would substitute for the gene knockout query line that could be screened through direct-tests, cross-species approaches, or whole genome approaches to uncover candidate synthetic dosage lethal interactors. Furthermore, each of these approaches would be expected to have similar caveats to those described above. For example, the ability to identify *bona fide* interactors will depend on the silencing efficacy of the siRNA/shRNA employed and overall gene coverage within the RNAi-based libraries. In any case, as synthetic dosage lethal interactors are identified, it will be essential to validate the initial findings prior to initiating any pre-clinical or clinical studies.

## 3. Conclusions—Synthetic Genetic Approaches in a Personalized Medicine Era

Novel therapeutic strategies and targets are required to not only decrease mortality rates associated with cancer, but to better focus the therapy to minimize side effects as well. Synthetic genetic approaches represent a paradigm shift in how tumors can be killed. These strategies differ from many traditional approaches in that it not only distinguishes tumor cells from normal surrounding tissues, but the genetic defects also serve as molecular beacons to restrict the cytotoxic effects to tumor cells. Synthetic lethality and synthetic dosage lethality are designed to specifically kill cancer cells based on the loss-of-functions associated with tumor suppressor genes or the gain-of-functions associated with oncogenes, respectively. Both approaches have been applied in cell-based screens and numerous, novel candidate drug targets have been identified. In either approach, the penultimate goal of these screens is to identify candidates for which small molecule inhibitors will ultimately be identified or developed. Presumably, once a target is identified an appropriate chemical screen can be devised to identify lead chemical compounds for subsequent validation, optimization and pre-clinical studies, prior to ultimately initiating clinical trials (Figure 3). Depending on the target identified and the availability of protein structural information, it may be possible to perform *in silico* docking experiments to identify candidate small molecule inhibitors (reviewed in [116,117,118]). Alternatively, chemical screens can be devised and performed to identify lead chemical compounds for subsequent validation and optimization studies [53], or alternatively, if small molecule inhibitors have been approved for human use, clinical studies could be initiated. In any case, most candidate compounds will require extensive testing and target validation in pre-clinical models prior to entering human clinical trials.

Tumor heterogeneity and the development of drug resistance to current therapeutic approaches make combating cancer a difficult, but not impossible task. With the advancement of next generation sequencing platforms and decreases in associated costs, it may eventually become possible to tailor cancer treatments to individuals rather than the disease type. For example, next generation sequencing [20,22,24,25,26,115,119] and chromatin immunoprecipitation sequencing (ChIP-Seq; reviewed in [120]) are already being performed on cancer cell lines and patient samples and can provide extensive information and a global view of the types of somatic mutations and epigenetic changes associated with a given tumor [121,122,123]. Based on this information, it may be possible to identify a unique combination of synthetic genetic targets and derive an appropriate combination therapy that selectively exploit the genetic and epigenetic defects in tumor suppressor genes and oncogenes to better combat the disease and minimize adverse side effects. However, this concept of personalized medicine is still in its infancy as synthetic lethal and synthetic dosage lethal interactors are still being identified, and potent and specific inhibitors will still have to be developed. Nevertheless, a large number of pre-clinical and clinical studies are now underway to evaluate the efficiency of synthetic lethal and synthetic dosage lethal approaches. Thus, synthetic genetic approaches may hold tremendous potential as a novel paradigm in combating cancers by selectively targeting the genetic basis of tumor development and progression.
